# Endemic Goitre

**Published:** 1906-05-05

**Authors:** 


### ENDEMIC GOITRE.

In a recent communication to the Royal Medical and Chirurgical Society[1] Dr. R. McCarrison gave the results of three years' observations carried on in two districts of Northern India. Of these the first was Chitral, a district presenting the following features. It is a mountainous country, whose villages are situated on alluvial fans at the mouths of nullahs. The rocks are everywhere metamorphic, gneiss and slate predominating; but the most goitrous villages are invariably associated with limestone outcrops. The climate is temperate and the rainfall small. The people are poor, their food being entirely vegetable and consisting of the poorest grains. Fresh meat and even salt are unknown except to the richest among them. The water supply is in all cases derived from the melting of the snows on the hills above. It runs down the nullahs as a turbulent mountain stream, taking up matter in its course both in solution and suspension. In the summer months the water is always grey from the presence of a fine suspension. There are no real glaciers in the district. The villages have no wells, and, owing to the sloping of the ground and the nature of the soil, water cannot stagnate. An analysis of the water supply showed that it varied largely in composition for the various villages, that no single dissolved ingredient appeared to have any influence in the production of goitre; nor did the total solids or the total hardness of the water seem to dictate the frequency of the disease.

The author states that in this district goitre appears among children of all ages, even among children at the breast. Females also are more liable to the disease than males. As regards the length of time necessary for the establishment of the malady, he found that in one instance 18 per cent. of susceptible individuals, natives of non-goitrous areas, developed the disease four months after the commencement of residence in one of the most goitrous villages. In another instance of ten young men from the plains of India two developed goitre in two and a half months after entering a goitrous zone. It is further stated that of all the male children in three of the most goitrous villages who were less than one year of age, no less than 32 per cent. showed evidence of the disease. Some of these cases may, in the author's opinion, be congenital, but some are no doubt acquired. Symmetrical goitres, except in long-standing cases, diminish in size when the subject leaves the area where the disease was acquired, but recover their size on his return. This rule appears to apply even when the new district is a goitrous one, if sufficient time has not elapsed for him to come under the new influence. Experiments were conducted with a view to testing the effect of boiling and filtering waters held to be capable of producing the disease. Ten dogs were kept in extremely goitrous houses for two months, five being given boiled water and five ordinary water. One of the latter five developed the disease.

The second series of observations was conducted in Gilgit and Nagar, districts whose geographical, physical, and social conditions are similar to those of Chitral. The water comes from a single source, and is conveyed to the villages in open channels. This water is capable of producing 11.8 per cent. of goitre at its source, while at its terminus, after serving as drinking supply and open sewer to many villages, it causes the disease to the extent of 45.6 per cent. One village in the district, however, which derives its supply from a small spring, is entirely free from the disease. This exemption proves the water-borne nature of the malady in the other parts of Gilgit. Analytical and microscopic examination of the water at different points in its passage from the source show that the dissolved ingredients do not vary appreciably, while the nearer the water is to its source the less suspended matter and the more diatoms does it contain. As regards the factors at work in the production of goitre, there are three possibilities. It might, theoretically, be due to an increase in the amount of some dissolved ingredient, or to an increase in the amount of suspended matter, whether organic or inorganic, or to micro-organismal pollution. The author considers the explanation to lie in the addition of some infective organism. The information afforded by the history of goitre in Nagar is very striking. Until six years ago Nagar was an independent state, cut off by inter-tribal jealousies from all communication with goitrous districts. Some five years ago certain goitrous individuals settled in the country, where previously goitre had never been known to originate, and now throughout Nagar goitre is spreading in a typically epidemic form, its first victims being children under ten years of age. The water supply of the affected area of Nagar is derived from a spring which is held to have existed from time immemorial. Its composition is therefore not likely to have undergone any spontaneous change. Nothing in the circumstances of the people has altered, but goitre has been introduced from without. "It seems likely, therefore, that some poison, goitre-producing in its powers, has been introduced into a water supply which happened to be suitable for the conveyance of the disease." Dr. McCarrison is of opinion that the state of affairs in Nagar is only explicable on the assumption that the causative factor is an organism. This hypothesis he supports by the following considerations: (1) The large percentage of goitrous children under one year of age in Chitral can be explained in this way without recourse to the conclusion that such cases are congenital; (2) on the infective hypothesis it is possible to account for such instances of the disease as cannot be traced to the water supply; (3) this hypothesis explains the regular increase in the frequency of the disease from the source to the terminus of the Gilgit water supply.

[1] April 10, 1906.

